# Case Study of Two Post Vaccination SARS-CoV-2 Infections with P1 Variants in CoronaVac Vaccinees in Brazil

**DOI:** 10.3390/v13071237

**Published:** 2021-06-26

**Authors:** Cassia F. Estofolete, Cecilia A. Banho, Guilherme R. F. Campos, Beatriz de C. Marques, Livia Sacchetto, Leila S. Ullmann, Fabio S. Possebon, Luana F. Machado, Juliana D. Syrio, João P. Araújo Junior, Cintia Bittar, Paula Rahal, Suzana M. A. Lobo, Helena Lage Ferreira, Nikos Vasilakis, Mauricio L. Nogueira

**Affiliations:** 1 Laboratório de Pesquisas em Virologia (LPV), Medicine School of São José do Rio Preto (FAMERP), São José do Rio Preto, São Paulo 15090-000, Brazil; cassiafestofolete@gmail.com (C.F.E.); ceci.abanho@gmail.com (C.A.B.); guilhermecampos07@gmail.com (G.R.F.C.); bbiacarvalho@outlook.com (B.d.C.M.); liviasacchetto@gmail.com (L.S.); 2 Hospital de Base, São José do Rio Preto, São Paulo 15090-000, Brazil; luana_famema@yahoo.com.br (L.F.M.); jdsyrio@yahoo.com.br (J.D.S.); suzanaalobo@gmail.com (S.M.A.L.); 3 Biotecnology Institute, São Paulo State University (UNESP), Botucatu, São Paulo 18607-440, Brazil; leila.ullmann@unesp.br (L.S.U.); fabio.possebon@unesp.br (F.S.P.); joao.pessoa@unesp.br (J.P.A.J.); 4 Intitute of Biosciences, Languages and Exact Sciences, São Paulo State University (UNESP), São José do Rio Preto, São Paulo 15054-000, Brazil; cibittar@gmail.com (C.B.); p.rahal@unesp.br (P.R.); 5 Department of Veterinary Medicine FZEA-USP, University of São Paulo (USP), Pirassununga, São Paulo 13635-900, Brazil; hlage@usp.br; 6 Department of Pathology, University of Texas Medical Branch, 301 University Blvd., Galveston, TX 77555, USA; nivasila@utmb.edu; 7 Sealy Center for Vector-Borne and Zoonotic Diseases, University of Texas Medical Branch, 301 University Blvd., Galveston, TX 77555, USA; 8 Center for Biodefense and Emerging Infectious Diseases, University of Texas Medical Branch, 301 University Blvd., Galveston, TX 77555, USA; 9 Center for Tropical Diseases, University of Texas Medical Branch, 301 University Blvd., Galveston, TX 77555, USA; 10 Institute for Human Infection and Immunity, University of Texas Medical Branch, 301 University Blvd., Galveston, TX 77555, USA

**Keywords:** SARS-CoV-2, COVID-19, vaccine

## Abstract

The rapid development of efficacious and safe vaccines against coronavirus disease 2019 (COVID-19) has been instrumental in mitigating the transmission of severe acute respiratory syndrome coronavirus 2 (SARS-CoV-2). Moreover, the emergence of SARS-CoV-2 variants raised concerns on the efficacy of these vaccines. Herein, we report two cases of breakthrough infections with the P1 variant in patients vaccinated with CoronaVac, which is one of the two vaccines authorized for emergency use in the Brazilian immunization program. Our observations suggest that the vaccine reduced the severity of the disease and highlight the potential risk of illness following vaccination and subsequent infection with the P1 variant as well as for continued efforts to prevent and diagnose infection in vaccinated persons.

## 1. Introduction

Over the past 12 months of the unprecedented COVID-19 pandemic characterized by high morbidity and mortality rates, the development of vaccines capable of containing COVID-19 has become a global priority. In Brazil alone, as of 25 April 2021, 14,237,078 individuals were infected by SARS-CoV-2 with 386,416 deaths. Globally, there are 146,067,511 recorded infections with 3,092,497 deaths [1]. According to the World Health Organization (WHO), as of 9 April 2021, 96 vaccines were in various stages of clinical development [2]. Currently, the CoronaVac (Butantan Institute, São Paulo, Brazil and Sinovac Life Sciences, Beijing, China) is one of the vaccines that has received emergency use authorization (EUA) by Agência Nacional de Vigilância Sanitária (ANVISA) for use in Brazil, with efficacy rates against symptomatic COVID-19 of 50.7% and symptomatic disease requiring assistance at 50.7% and 83.7%, respectively [3].

However, throughout the duration of CoronaVac’s clinical trial, the unrestrained SARS-CoV-2 transmission and spread has allowed the emergence of SARS-CoV-2 variants, which are classified as Variants of High Consequence (VOHC), Variants of Interest (VOI), and Variants of Concern (VOC). Variants of Concern are considered those with increased transmissibility and severity as well as significant reduction in the neutralization capacity by antibodies generated in the course of previous infection or vaccination [4]. Three VOCs have been currently circulating in Brazil: British B.1.1.7, South African B.1.351, and the Brazilian B.1.1.28 or P1 [5], whose emergence has raised concerns of the efficacy of the COVID-19 vaccines. In the United States, as of 30 April 2021, 10,262 SARS-CoV-2 vaccine breakthrough infections had been reported out of nearly 101 million fully vaccinated persons (0.0001%), of which 2725 (27%) were asymptomatic, 995 (10%) required hospitalization, and 160 (2%) were fatal [6]. Herein, we describe the clinical outcomes of breakthrough infections with the P1 variant in two patients vaccinated with CoronaVac.

## 2. Materials and Methods

This study describes the two cases of patients vaccinated against SARS-CoV-2 with CoronaVac, who required ventilation support during their hospitalization following SARS-CoV-2 infection. Both patients were enrolled among 651 participants in the stage 3 clinical trial of CoronaVac, which was carried out by the Butantan Institute in 16 Brazilian centers with 12,396 participants enrolled between 21 July and 16 December 2020 (ClinicalTrials.gov, accessed on 25 June 2021; Identifier: NCT0445659). Unblinding of the vaccine trial’s results took place approximately one month prior to their hospital admission, confirming the enrollment of both patients in the vaccine group.

Epidemiological and clinical data (symptoms and radiologic observations) were obtained from electronic records. Naso/oropharyngeal samples were submitted for further diagnostic tests at the Laboratório de Pesquisas em Virologia (LPV), which is located within the Faculdade de Medicina de São José do Rio Preto (FAMERP). This study was submitted and approved by the Ethical Review Board of the Faculdade de Medicina de São José do Rio Preto (FAMERP), São Paulo, Brazil (protocol 34634620.1.2003.5415, 14 July 2020).

Viral RNA was extracted from 140 ul of nasal swabs using QIAamp Viral RNA Mini Kit (QIAGEN, Hilden, Germany), following the manufacturer’s instructions. One-step real-time polymerase chain reaction (RT-qPCR) was performed using primers and probes targeting the envelope (E) and nucleocapsid (N) of the SARS-CoV-2 genome and human RNAse P designed GeneFinder COVID-19 PLUS RealAmp Kit (OSANG Healthcare Co., Ltd, Gyeonggi-do, Korea). Variant tracking was achieved by the amplification of a 798 bp fragment from the Spike genomic region that includes the Receptor Binding Domain (RBD) by conventional PCR using a set of specific primers (Forward SARS-CoV2_S1_PF: 5′-GAGTCCAACCAACAGAATC-3′ and Reverse SARS-CoV-2_S1_PR: 5′-GAATCTCAAGTGTCTGTGG-3′) and Sanger sequencing using BigDye Terminator v.3.1 (Applied Biosystems, Waltham, MA, USA). Readings were performed in a 3130xl Genetic Analyzer (Applied Biosystems, Waltham, MA, USA), contigs were assembled using the Electropherogram Quality Analysis tool (http://asparagin.cenargen.embrapa.br/phph/, accessed on 17 April 2021), and sequences were aligned using Clustal W [7] available in the BioEdit package [8]. Complete genomic sequences of the variants were obtained by next-generation sequencing. Briefly, library preparations were carried out following the instructions provided by an Illumina CovidSeq Test (Illumina Inc, San Diego, CA, USA). The quality and size of the libraries were verified by Agilent 4150 TapeStation (Agilent Technologies Inc, Santa Clara, CA, USA). Libraries were pooled in equimolar concentrations, and the sequencing was implemented on an Illumina MiSeq system, using a MiSeq Reagent Kit v2 (2 × 150 cycles) (Illumina Inc, San Diego, CA, USA). The quality of FASTQ sequencing data was checked using FastQC software (http://www.bioinformatics.babraham.ac.uk/projects/fastqc, accessed on 25 April 2021), and trimming was performed in Geneious Prime v. 2021.1, using the software BBDuk v. 37.25, in order to remove adapters and low-quality bases. A minimum Phred score of Q30 and minimal read length of 75 base pairs (bp) were used. The cleaned paired-end reads were mapped against the hCoV-19/Wuhan/WIV04/2019 (EPI_ISL_402124) genome, which is available in the EpiCov database in GISAID (https://www.gisaid.org/, accessed on 7 May 2021), considering a minimum 50 bp overlap, minimum identity of 90%, and maximum mismatches of 10% per read. SNPs were identified using default settings and minimum coverage of 5 reads per site. The genomes of the both samples under study were aligned against the previous sequences obtained by Sanger sequencing in order to confirm the mutations sites and then submitted to Pangolin COVID-19 Lineage Assigner Tool to confirm the variant classification [9]. Finally, the phylogenetic analysis was performed to support the results of lineage assignment. Consensus sequences (hCoV-19/Brazil/SP-SJRP14/2021, GISAID access EPI_ISL_1754186 and hCoV-19/Brazil/SP-SJRP15/2021, GISAID access EPI_ISL_1941583) were aligned with 103 SARS-CoV-2 complete genomes from Brazil, retrieved from GISAID (Table S1) and the reference genome hCoV-19/Wuhan/WIV04/2019 (EPI_ISL_402124). Sequencing alignment was carried out in MAFFT multiple alignment software version 7.271 [10] with default parameters. A maximum-likelihood tree was built in IQ-TREE (v. 2.1.3), using GTR+F+I as the nucleotide substitution model and 1000 bootstrap samples.

## 3. Results

### 3.1. Case 1

A 60-year-old man with a history of diabetes mellitus type 2, high blood pressure, and obesity degree I (BMI: 32.3 kg/m^2^), who was a resident of São José do Rio Preto, São Paulo State, Brazil, and no recent travel history, reported on 10 February 2021 with anosmia, malaise, and myalgia. He had been vaccinated with two doses of CoronaVac through enrolment in a clinical trial developed by the Butantan Institute in October 2020 (first dose: 8 October, second dose: 27 October; diagnosis of breakthrough infection 106 days following administration of the 2nd vaccine dose). Unblinding of the trial was in January 2021, which confirmed his enrollment in the vaccine group.

**Figure 1:**
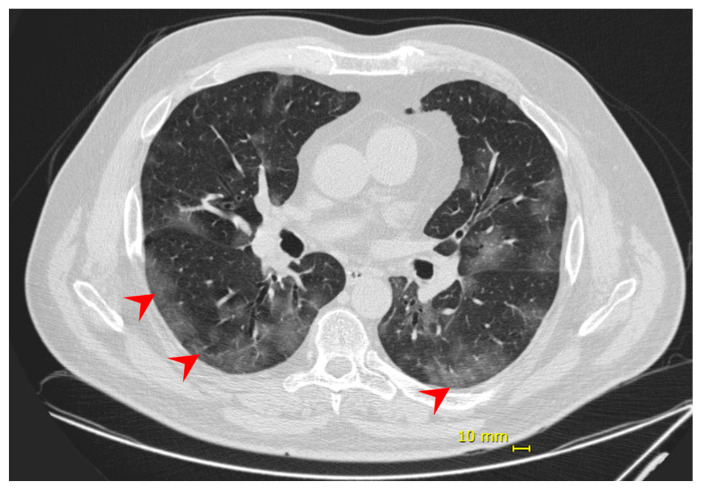
Patient 1—chest computed tomography evidenced bilateral multifocal ground-glass opacities (25–50%) predominantly peripheral (red arrow).

Eight days following the onset of symptoms, the patient complained of dyspnea and decreased peripheral saturation of oxygen (SpO2). He was admitted at the hospital with blood pressure 125/85 mmHg; heart rate 98 bpm; respiratory rate 18 ipm; temperature 35.1 °C; and SpO2 88% without supplemental oxygen. No additional alterations were observed in the physical exam. Chest computed tomography (chest CT) showed evidence of bilateral multifocal ground-glass opacities (25–50%) predominantly peripheral and subsegmental atelectasis (Figure 1), and COVID-19 diagnosis was confirmed by a positive RT-PCR on the submitted nasal/oropharyngeal swab sample. The patient was monitored in the intensive care unit (ICU) and received supplemental oxygen by high flow nasal catheter until one day before the discharge. No intubation or other ventilation device was needed. During hospitalization, the patient also received dexamethasone 6 mg, once a day, for 10 days, and enoxaparin 40 mg, as a prophylaxis regimen. He was discharged 12 days from hospital admission and 20 days from symptoms onset, having fully recovered.

### 3.2. Case 2

On 26 February 2021, a 55-year-old male resident of São José do Rio Preto, São Paulo State, Brazil, without comorbidities, BMI: 25.9 kg/m^2^, was admitted at our hospital complaining of sore throat, headache, malaise, chills, coryza, and sneezing. A COVID-19 diagnosis was confirmed by a positive RT-PCR on the submitted nasal/oropharyngeal swab sample. He had a recent trip to Espírito Santo State, Brazil, returning 4 days before the onset of symptoms. He had been vaccinated with two doses of CoronaVac in October 2020 (first dose: 9 October, second dose: 27 October; diagnosis of breakthrough infection 122 days following administration of the 2nd vaccine dose) through enrolment in a clinical trial developed by the Butantan Institute. Unblinding of the trial in January 2021 confirmed his enrollment in the vaccine group.

**Figure 2:**
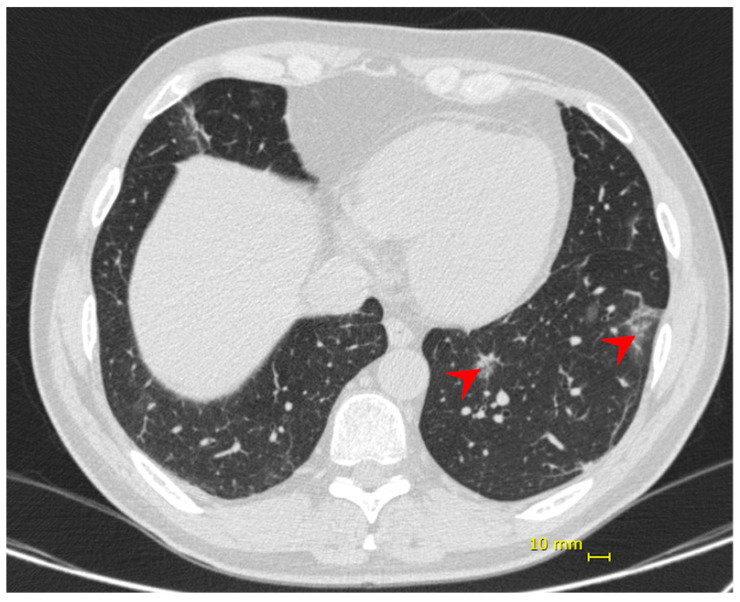
Patient 2—chest computed tomography showing bilateral multifocal ground-glass opacities (<25%) predominantly peripheral (red arrow).

Nine days following the onset of symptoms, the patient presented dyspnea and hypoxia (SpO2 84% without supplemental oxygen), blood pressure 97/54 mmHg, heart rate 86 bpm, respiratory rate 31 ipm, temperature 36.9 °C. No additional alterations were observed in the physical exam. Chest CT showed bilateral multifocal ground-glass opacities (<25%) predominantly peripheral and subsegmental atelectasis (Figure 2). He received supplemental oxygen by high-flow nasal catheter, dexamethasone 6 mg, once a day, for 10 days, and enoxaparin 40 mg as prophylactic dose. His discharge took place 6 days from his hospital admission and 14 days from the onset of symptoms.

The clinical and laboratory results of both cases are presented below in Table 1. As in other studies with severe cases (reviewed by [11,12]), lymphocytopenia, increased lactate dehydrogenase, and C-reactive protein were observed.

### 3.3. Variant Tracking

**Figure 3:**
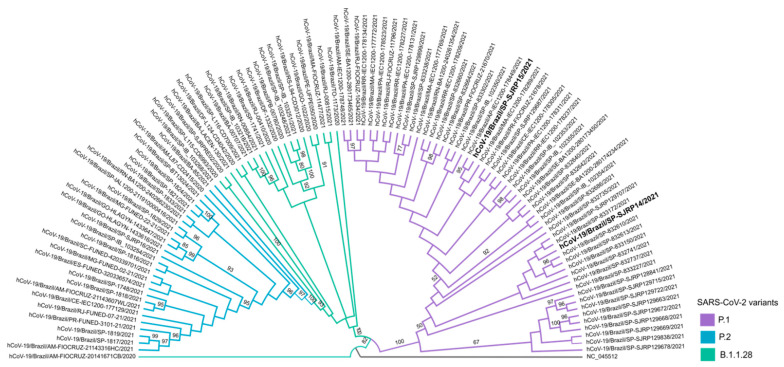
Phylogenetic relationship of hCoV-19/Brazil/SP-SJRP14/2021 and hCoV-19/Brazil/SP-SJRP15/2021 (in bold). Maximum-likelihood tree was built in IQ-TREE (v. 2.1.3), using GTR+F+I as a nucleotide substitution model. Bootstrap: 1000.

Naso/oropharyngeal samples were submitted for additional tests for variant tracking. The samples of both patients were designated hCoV-19/Brazil/SP-SJRP14/2021 and hCoV-19/Brazil/SP-SJRP15/2021, respectively, and classified as P1 lineage by the Pango lineage assignment tool, as well as by phylogenetic inference with high branch support (Figure 3). Sequence analysis showed that hCoV-19/Brazil/SP-SJRP14/2021 exhibited 13 non-synonymous mutations in the Spike protein. From these changes, ten are defining SNPs of the P1 variant (aa:S:L18F, aa:S:T20N, aa:S:P26S, aa:S:D138Y, aa:S:R190S, aa:S:K417T, aa:S:E484K, aa:S:N501Y, aa:S:H655Y and aa:S:T1027I), and three are amino acid substitutions (S:N196Y, S:D614G, S:V1176F) that have been reported in other lineages, such as B.1.1.28, from which P1 is derived. Additionally, several amino acid changes were also verified in other genome regions (Table 2). The hCoV-19/Brazil/SP-SJRP15/2021 displayed ten non-synonymous substitutions in the Spike protein; from those, nine are well characterized as SNPs from the P1 variant. Moreover, several amino acid mutations were observed in the whole genome, as in hCoV-19/Brazil/SP-SJRP14/2021. The small number of mutations found in hCoV-19/Brazil/SP-SJRP15/2021 compared with hCoV-19/Brazil/SP-SJRP14/2021 is a consequence of less than optimal genome coverage.

## 4. Discussion

In December 2019, the first cases of an atypical pneumonia were described in individuals in China, and SARS-CoV-2 was identified as its etiologic agent [13]. The economic and social impact of the new disease, COVID-19, were unimaginable at the time. The virus spread first within China, from where it spread throughout the globe and was declared as a public health emergency of international concern by the World Health Organization (WHO) [14] on 11 March 2020 and right afterwards a pandemic [15]. The first documented case of COVID-19 in Brazil was reported in a Brazilian traveler in February 2020, who had returned from a trip to Lombardy, Italy [16]. Since then, the virus spread uncontrolled throughout the country, reaching 15,003,563 COVID-19-confirmed cases and 416,949 deaths as of 06 May 2021.

Manaus, the capital of Amazonas State, is the largest city in the Amazon, and it was the epicenter of attention for the dire consequences of SARS-CoV-2 in Brazil. The city reported an explosive number of cases at the beginning of the epidemic with high mortality rates [17]. Data showed that up to three-quarters of its population had been infected by SARS-CoV-2 in the first months of 2020, leading epidemiologists to suggest that herd immunity may have contained the epidemic [18,19]. However, the achieved high levels of community exposure were insufficient in preventing a second wave of COVID-19 cases, which was caused by the emergence of a new variant (P1) of SARS-CoV-2 [20], leading to the total collapse of the local health system [21].

Soon, the P1 variant (as well as other variants) spread in many Brazilian states [22], including São Paulo, where São José do Rio Preto is located. P1 is a variant that raised great concern since it is associated with higher transmission and infection rates as well as a reduction in antibody-mediated immunity (reviewed in [23]), which were characteristics attributed to a set of mutations (K417T, E484K, N501Y) in the RBD region of the S protein [24,25] and D614G [26]. The combination of these spike mutations, which is also shared with the B.1.351 variant, has been implicated in the decrease of neutralizing activity, suggesting that neutralizing antibodies are less effective against VOCs with this set of mutations [27,28,29,30]. That led to the hypothesis SARS-CoV-2 VOCs may produce more severe disease and be poorly protected against post-vaccination [31,32]. Herein, we described two cases of breakthrough infections in two CoronaVac-vaccinated individuals that were infected by the P1 SARS-CoV-2 variant and had favorable clinical outcomes.

The concern of vaccine efficacy against variants was raised early in the establishment of the national program of COVID-19 immunization, leading to the development and implementation of step-by-step protocols in diagnosing their circulation and effect on the vaccine efficacy among the population [33]. Such concern has been given more attention as several mutations were detected in spike glycoprotein. Many vaccines against COVID-19 are based on a version of the spike glycoprotein [reviewed in [34]] to inducing neutralizing antibodies, and since that site is the main target of mutations, the immune escape is unclear.

Despite the unprecedented and uncontrolled spread of the virus in Brazil, the rapid approval and vaccine immunization drives among the population offer a growing sense of relief in controlling the epidemic. The safety and immunogenicity of CoronaVac, one of the vaccines administrated in Brazil, had been already presented in a recent phase 1/2 clinical trial in adults [35], and its efficacy data were shown in a randomized, double-blind, placebo-controlled phase 3 clinical trial developed in Brazil [3]. Additional evidence for its efficacy in a high P1 variant transmission setting was recently presented. In this case-control study, vaccination with at least one dose resulted in a 0.50-fold reduction of symptomatic SARS-CoV-2 infection (adjusted vaccine effectiveness, 49.6%; 95% CI, 11.3–71.4), underscoring the need for increase in the vaccination efforts [data not peer reviewed yet [36]]. As of 3 May 2021, 14.05% of the Brazilian population had received at least one vaccine dose, and 6.63% were fully vaccinated [37].

Our study reports the clinical outcomes in two CoronaVac vaccinated patients who acquired a breakthrough SARS-COV-2 infection. Although both patients required hos-pitalization, the disease presentation was mild and did not progress in severity requiring invasive mechanical ventilation (IMV) or death. We believe that their quick recovery and absence sequelae were directly related to their recent immunization against COVID-19, as has been previously suggested [38]. Although the vaccine did not prevent SARS-CoV-2 infection by the P1 variant, there was no need for IMV, and both patients were recovered at discharge. In a retrospective study based on the first 250,000 COVID-19 hospitalizations in Brazil, the rates of IMV and death were 23% and 38%, respectively [39]. An important limitation of our study was a lack of serological, cytokine or cellular data to account for their re-infection. However, caution should be exercised in inferences derived from serological assays for the protective efficacy of SARS-CoV-2 vaccination against VOCs, as the correlates of protection are still not determined. The outcomes suggest that CoronaVac may be contributing to the reduction of severe clinical outcomes in SARS-COV-2 P.1 infections [35].

## Figures and Tables

**Table 1:** Clinical and laboratory findings during the progressing of COVID-19 in two patients vaccinated with CoronaVac. In bold: out of range results from reference values.

	Patient 1			Patient 2				
**Signs and symptoms**	Anosmia, malaise, and myalgia			Sore throat, headache, coryza, malaise, chills, sneezing				
**Worsening day**	D8-dyspnea and SpO2 88%			D9-dyspnea and SpO2 84%				
**Clinical management**	Supplemental oxygen by high flow nasal catheter. Dexamethasone and enoxaparin as prophylactic dose			Supplemental oxygen by high flow nasal catheter. Dexamethasone and enoxaparin as prophylactic dose				
**Chest CT**	Bilateral multifocal ground-glass opacities (25–50%) predominantly peripheral and subsegmental atelectasis			Bilateral multifocal ground-glass opacities (<25%) predominantly peripheral, and subsegmental atelectasis				
**Discharge**	Recovery, 20 days from symptoms onset			Recovery, 14 days from symptoms onset				
**Laboratory findings**	**Admission**	**Recovery**	**Discharge**	**Admission**	**Clinical Worsening**	**Recovery**	**Discharge**	**Reference values**
Hemoglobin (g/dL)	**17.7**	15.6	15.3	13.3	13.8	13.2	13.2	12–16
Hematocrit (%)	**51.5**	44.2	43.9	39.1	39.8	39.8	44.3	36–47
Leukocytes (cells/mm^3^)	5880	7400	7020	5150	7160	8990	**11,200**	4–11 × 10^3^
Neutrophils (cells/mm^3^)	4720	5790	4090	4630	5580	6837	7470	1.6–7.7 × 10^3^
Lymphocytes (cells/mm^3^)	730	760	1840	780	860	899	1650	0.6–3.9 × 10^3^
Platelets (×10^3^)	**120**	322	327	**123**	**125**	168	214	150–450 × 10^3^
CRP (mg/dL)	**5.14**	**1.42**	0.16	**1.96**	**9.8**	**2.27**	**1.99**	up to 0.50
LDH (U/L)	**393**	**393**	**317**	178	**324**	**288**	**287**	1–250
Dimer D (ug/mL)	0.42	0.34	0.33	0.34	**1.26**	**0.97**	**0.7**	up to 0.500
AST (U/L)	**46**			18	**26**	**106**		15–24
ALT (U/L)	**36**			**28**	16	**185**		15–24
GGT (U/L)	67			29	34	**173**		up to 123
AP (U/L)				65		75		Up to 104
Creatinine (mg/dL)	0.7	0.6	0.7	**1.3**	0.9	0.9	1.1	0.7–1.2
pH	7.44	**7.49**	7.46	7.46	7.43	7.42		7.35–7.45
PO2 (mmHg)	**69.7**	84.6	83.8	109	80.3	**69.8**		80–100
PCO2 (mmHg)	**35.8**	**34.7**	**34.9**	**33.7**	**34.6**	41.1		38–50
HCO3 (mmol/L)	23.9	26	24.5	23.8	22.6	26.4		22–26
EB (mmol/L)	1.1	1.3	1.9	0.8	−0.6	**2.3**		−2.0–2.0
SatO2 (%)	**91.6**	95.1	**93.9**	98	97.6	**93.9**		94–100
Lactate (mmol/L)	**4.1**	**1.7**	1.6	1.3	1.5	**2.3**		0.5–1.6

**Table 2:** Non-synonymous changes identified in the whole-genomes of hCoV-19/Brazil/SP-SJRP14/2021(EPI_ISL_1754186) and hCoV-19/Brazil/SP-SJRP15/2021 (EPI_ISL_1941583). In bold: mutations of concern.

Samples	ORF1a	ORF1b	S	ORF3a	M	ORF8	ORF9b	N
hCoV-19/Brazil/SP-SJRP14/2021	ORF1a:S1188L, ORF1a:K1795Q, ORF1a:S3675-, ORF1a:G3676-, ORF1a:F3677-	ORF1b:P314L, ORF1b:E1264D	S:L18F, S:T20N, S:P26S, S:D138Y, S:R190S, S:N196Y, **S:K417T**, **S:E484K**, **S:N501Y**, S:D614G, S:H655Y, S:T1027I, S:V1176F	ORF3a:S253P	−	ORF8:E92K	ORF9b:Q77E	N:P80R, N:R203K, N:G204R
hCoV-19/Brazil/SP-SJRP15/2021	ORF1a:K1795Q	ORF1b:P314L, ORF1b:E1264D	S:L18F, S:T20N, S:D138Y, S:R190S, **S:K417T**, **S:E484K**, **S:N501Y**, S:D614G, S:H655Y, S:T1027I	ORF3a:L94F, ORF3a:S253P	M:T7N, M:I8S	ORF8:E92K	ORF9b:Q77E	N:P80R

## Data Availability

All SARS-CoV-2 genomes generated and analyzed in this study are available in the EpiCov database in GISAID at: https://www.gisaid.org/, accessed on 25 June 2021. Accession IDs are listed in Table S1.

## Supplementary Materials

The following are available online at https://www.mdpi.com/article/10.3390/v13071237/s1, Table S1: Information of SARS-CoV-2 sequences included in the dataset.
